# American Cutaneous Leishmaniasis: Imported cases in Berlin 2000–2023

**DOI:** 10.1371/journal.pntd.0012323

**Published:** 2024-07-15

**Authors:** Andreas K. Lindner, Maria Cristina Moreno-del Castillo, Mia Wintel, Gabriela Equihua Martinez, Joachim Richter, Florian Kurth, Frieder Pfäfflin, Thomas Zoller, Maximilian Gertler, Susanne Georgi, Michael Nürnberg, Claudia Hülso, Julian Bernhard, Sarah Konopelska Kotsias, Antonio Seigerschmidt, Welmoed van Loon, Frank Mockenhaupt, Beate Kampmann, Gundel Harms

**Affiliations:** 1 Charité–Universitätsmedizin Berlin, corporate member of Freie Universität Berlin and Humboldt-Universität zu Berlin, Charité Center for Global Health, Institute of International Health, Berlin, Germany; 2 Charité–Universitätsmedizin Berlin, corporate member of Freie Universität Berlin and Humboldt-Universität zu Berlin, Department of Infectious Diseases, Respiratory and Critical Care Medicine, Berlin, Germany; Universidade de São Paulo: Universidade de Sao Paulo, BRAZIL

## Abstract

**Background:**

American Cutaneous Leishmaniasis (ACL) shows variable response to therapy, but data on species-specific treatment efficacy is scarce. We describe the clinical characteristics and outcome of patients with ACL imported to a tertiary centre in Germany and determine whether species-specific therapy according to the 2014 “LeishMan” group recommendations is associated with cure.

**Methods:**

A retrospective chart review was conducted at the Charité Institute of International Health in Berlin. We analysed data on PCR-confirmed ACL cases collected between 2000 and 2023. Systemic therapy included liposomal amphotericin B, miltefosine, pentavalent antimony, ketoconazole or itraconazole. Localized therapy included perilesional pentavalent antimony or paromomycin ointment. Cure was defined as re-epithelialization of ulcers or disappearance of papular-nodular lesions after 3 months of treatment. Logistic regression models were used to quantify the effect of species-specific systemic therapy on the outcome.

**Results:**

75 cases were analysed. Most patients were male (62%), median age was 35 years, no patient had a history of immunosuppression. The most common reason for travel was tourism (60%), the most common destination was Costa Rica (28%), the median duration of illness was 8 weeks, and most patients presented with ulcers (87%). Lesions were complex in 43%. The most common *Leishmania* (L.) species was *L*. *braziliensis* (28%), followed by *L*. *panamensis* (21%). 51/73 (70%) patients were cured after initial therapy and 17/21 (81%) after secondary therapy. Cure after systemic therapy was more frequent when species-specific treatment recommendations were followed (33/45; 73%), compared to when not followed, (6/17; 35%, P = 0.008). This association was independent of age, sex, previous therapy, complex lesions, and *Leishmania* species (adjusted OR, 5.06; 95% CI, 1.22–24.16).

**Conclusions:**

ACL is a rare, imported disease in Germany. Complex lesions were common, challenging successful therapy. This study highlights the importance of identifying the parasite species and suggests that a species-specific approach to treatment leads to better outcomes.

## Author summary

American cutaneous leishmaniasis is a skin disease caused by parasites that are transmitted through the bite of sandflies. It is considered as a neglected tropical disease. In non-endemic countries like Germany, this disease is rarely seen and is imported by migrants or travellers who have been infected in tropical or subtropical areas of the Americas. Experts suggest that the treatment should be given depending on the specific parasite species found in the affected skin, but there is still a scarcity of information on the optimal management strategy. We analysed 75 American cutaneous leishmaniasis cases seen between 2000 and 2023 at our German medical centre. Most patients were men, around 35 years of age, who most commonly travelled to the Americas for tourism. We found that when using systemic medications (i.e., given by mouth, injection or drip into a vein), that were recommended for the specific parasite type, patients were more likely to be cured, with a 73% success rate, compared to a mere 35% success rate when those recommendations were not followed. This highlights the importance of identifying the species that is causing the disease, and suggests that tailoring treatment to the specific parasite species increases the chances of cure.

## Introduction

American Cutaneous Leishmaniasis (ACL) also known as New World Cutaneous Leishmaniasis, is a condition caused by various *Leishmania* protozoan species. It occurs in the American subtropical and tropical areas and is transmitted predominantly by infected *Lutzomyia* sandflies. The *Leishmania* genus is divided into the subgenera *Leishmania* and *Viannia*, each encompassing different *Leishmania* species. The *Leishmania* species have varying pathogenicity and drug susceptibility which also differs for the same species in different geographical regions [1,2].

Recognized by the WHO as a neglected tropical disease, cutaneous leishmaniasis (CL) remains a significant yet under prioritised global health issue [3]. It is estimated that around 1 million new cases of CL occur each year, though the exact number is unknown due to underreporting [4,5]. In 2022, 205,996 new cases of CL were reported globally from only 48 out of 90 endemic countries, and a mere 334 imported cases were reported, including 79 cases in Europe [6]. In non-endemic countries like Germany, the disease poses unique diagnostic and therapeutic challenges. There is a need for tailored clinical protocols and an enhanced understanding of ACL management in non-endemic contexts.

Therapeutic options for CL include the use of systemic drugs or localized therapy. The treatment approach for CL is determined by the clinical features, i.e., by the number, size, and localisation of the lesions, and by the presence or absence of mucosal and/or lymphatic involvement. Local therapy is preferred over systemic drugs whenever feasible (e.g., single or few lesions, not cosmetically disfiguring, lesions with diameter < 40 mm) to reduce the risk of systemic side effects, and to improve tolerability and cost-effectiveness [1,7,8]. Other factors to consider when choosing the therapy are the *Leishmania* species and the region of exposure. In recent years, *Leishmania* species-specific treatment guidelines for imported CL, e.g., by the Infectious Disease Society of America (IDSA) or the European “Leishman” expert group, replaced previous guidelines that were based on geographic exposure [1,8]. However, because leishmaniasis remains a neglected tropical disease, data on species-specific treatment efficacy of ACL is still limited. The goal of this retrospective analysis was to refine the understanding of clinical features, management and outcomes of imported ACL to our tertiary centre in Germany, as well as to assess whether adherence to the 2014 species-specific treatment recommendations of the European expert group “LeishMan” has an impact on patient outcomes.

## Methods

### Ethics statement

Ethical approval for using routinely collected data from patients with ACL was obtained from the ethics committee of Charité–Universitätsmedizin Berlin (EA2/224/22). According to the Berlin State Hospital Act, patient consent was not required for the retrospective evaluation of routine clinical data.

### Study population and setting

A retrospective study using a chart review methodology was conducted at the Charité Institute of International Health (IIH) in Berlin, Germany. Necessary data on demographics, exposure, clinical features, treatment and response were systematically extracted from an electronic healthcare database and/or from patients’ paper records. Data on laboratory confirmed ACL cases identified between 2000 and 2023 was collected. The patients presented either to the outpatient clinic of IIH or were referred to IIH from other medical facilities within Germany.

Ethical approval for using routinely collected data from patients with ACL was obtained from the ethics committee of Charité–Universitätsmedizin Berlin (EA2/224/22).

### Species-specific diagnostics

The diagnosis of ACL was made based on the patient’s travel history to endemic areas and the presence of characteristic skin and/or mucosal lesions, and then confirmed by polymerase chain reaction (PCR). For *Leishmania* species diagnosis, DNA was extracted from skin and/or mucosal punch biopsies, with scratch preparations for some individual cases. Sections of the gene coding for the ribosomal RNA, particularly the small subunit or internal transcribed spacer 1 region were amplified by PCR, followed by restriction fragment length polymorphism (RFLP) analysis [9]. Since 2011, *Leishmania* species were identified by PCR of the heat-shock protein 70 (HSP70) gene followed by RFLP analysis [10]. In cases where older preserved skin biopsies were accessible, retrospective species identification by HSP70-RFLP was performed.

### Treatment

Systemic therapy options included the use of intravenous (IV) liposomal amphotericin B (LAmB), oral miltefosine (available since 2008), IV or intramuscular pentavalent antimony (i.e. meglumine antimoniate), oral ketoconazole or itraconazole [1]. Localized therapy included perilesional infiltration with pentavalent antimony or paromomycin ointment [1]. After diagnostic confirmation, all patients except one received pharmacotherapy, and three patients had already started therapy before referral to our clinic.

### Definitions

Applying adapted classifications for disease severity used by the WHO/PAHO, IDSA and by the “LeishMan” expert group [1,7,8], patients were classified as having ≥ 4 lesions or < 4 lesions, presence or absence of lymphatic spread, and presence or absence of mucosal lesions. The main lesion was classified according to localisation, size in cm, and type as either an ulcer, papule or nodule, or other (e.g. plaque or crust). Complex lesions were defined by characteristics that have previously been linked to increased risk for mucosal spread (i.e., ≥ 4 skin lesions, at least one skin lesion > 4 cm, mucosal involvement or lymphadenopathy), other lesions were classified as simple lesions. We documented whether patients had received systemic therapy according to the 2014 “LeishMan group” recommendations [1].

We classified the *Leishmania* species that we identified based on their taxonomy at the subgenus, complex or species level, i.e., the *Leishmania* subgenus, which includes the *L*. *mexicana complex* with the species *L*. *mexicana* and *L*. *amazonensis*, and the *Viannia* subgenus which includes the *L*. *braziliensis complex* with the species *L*. *braziliensis* and *L*. *peruviana*; the *L*. *guyanensis complex* with the species *L*. *guyanenesis* and *L*. *panamensis*, and the *L*. *lainsoni complex* containing *L*. *lainsoni* species [11]. Outcomes were assessed after three months of treatment start. Healing or cure was defined as complete reepithelization of an ulcer or disappearance of a papular-nodular induration [12]. Partial remission was defined as < 100% but ≥ 50% reepithelization of an ulcer or regression of a papular-nodular lesion. Treatment failure was noted when reepithelization of an ulcer or regression of a papular-nodular induration was < 50%, or a new lesion appeared. Recurrence was defined as the reappearance of the ulcer or the induration after complete healing [12,13]. Adverse events were defined as any new symptom that was recorded after the medication was started. Severe adverse events were defined as any adverse event that posed an indication to stop the therapy.

### Statistical analysis

Statistical analysis included summary statistics, with continuous data presented as medians and interquartile ranges (IQR) and categorical variables presented as frequencies and percentages. Categorical variables were compared using Fisher’s exact test or Chi-square test as appropriate. We analysed the direct effect of species-specific therapy (as defined by the “LeishMan” group recommendations) [1] on healing after systemic therapy during a first or second therapeutic attempt. Potential confounders were selected *a priori* on the basis of clinical relevance and previous association with treatment outcomes [1,2,14–16]. Causal direct acyclic graphs (DAGs) [17] were constructed using DAGitty v.3.1 to identify the minimally sufficient confounding adjustment set (S1 Fig) [18]. Logistic regression models were constructed, and the effect was quantified using odds ratios (ORs) with 95% confidence intervals (CIs). Model 1 analysed the unadjusted association with species-specific therapy as independent variable and healing or cure as dependent variable. Model 2 additionally included age, sex, previous therapy, and “complex lesions” as a measure of disease severity (S1A Fig). Model 3 additionally included *Leishmania* species (S1B Fig). A two-tailed P-value of less than 5% was considered statistically significant for all tests. Statistical analysis was conducted using RStudio v.4.3.1.

## Results

**Fig 1:**
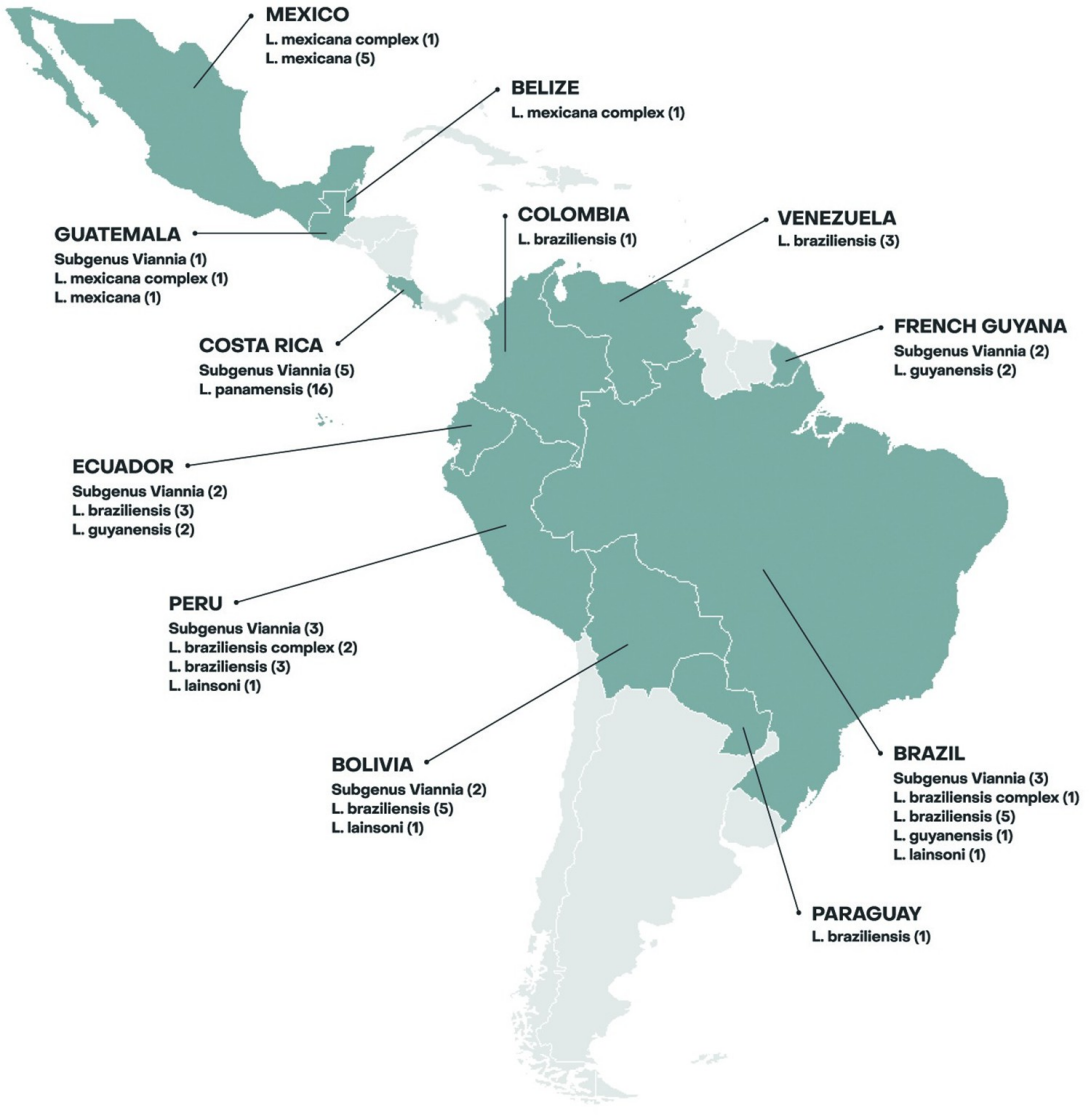
Geographical distribution according to the country of exposure and *Leishmania* species identified of the ACL cases imported to Berlin from 2000–2023. The map was created with the software program R using the following source of the basemap shapefile: Massicotte P, South A (2024). rnaturalearth: World Map Data from Natural Earth. R package version 1.0.1.9000, https://github.com/ropensci/rnaturalearth, https://docs.ropensci.org/rnaturalearthhires/, https://docs.ropensci.org/rnaturalearth/.

Data from 75 patients with ACL was analysed. Most patients were male (63%), median age was 35 years (IQR, 29–51; range, 12–70 years), no patient had known underlying immunosuppression, and most originated from Germany (80%). The most common reason for travel was tourism. The most common countries of exposure were Costa Rica (28%), Brazil (13%) and Peru (12%), see Fig 1. Clinical characteristics are shown in Table 1. 88 percent of infections were caused by species in the subgenus *Viannia*. Further differentiation to the species level was possible in 68%, with predominance of *L*. *braziliensis* (28%), followed by *L*. *panamensis* (21%).

**Table 1:** Characteristics of patients with imported ACL treated at IIH Berlin 2000–2023.

**Sex**	
Male	47 (63%)
Female	28 (37%)
**Age in years,** median (IQR)	35 (29–51)
**Reason for travel**	
Tourism	45 (60%)
Work or education	22 (29%)
Native or immigrant	7 (9%)
Unknown	1 (1%)
**Immunosuppression**	-
**Main lesion,** N = 74	
Ulcer	64 (87%)
Papule/ nodule	6 (8%)
Other	4 (5%)
**Main lesion size,** N = 62	
**≥ 4 cm**	9 (15%)
**< 4 cm**	53 (86%)
**Localisation of main lesion,** N = 73	
Face	21 (29%)
Arms and/or hands	20 (27%)
Legs and/or feet	23 (32%)
Trunk	6 (8%)
Several localisations	3 (4%)
**Lymphadenopathy/lymphatic spread**, N = 46	
Present	20 (44%)
Not present	26 (57%)
**Mucosal involvement**, N = 75	
Present	6 (8%)
Not present	69 (92%)
**Complex lesion**, N = 75	
Present	32 (43%)
Not present	43 (57%)
**Duration of illness**, median weeks (IQR), N = 65	8 (4–12)
**Leishmania species**, N = 75	
Subgenus Viannia *	18 (24%)
*L*. *braziliensis complex* *	3 (4%)
*L*. *braziliensis species*	21 (28%)
*L*. *guyanensis species*	5 (7%)
*L*. *panamensis species*	16 (21%)
*L*. *lainsoni species*	3 (4%)
*L*. mexicana complex*	3 (4%)
*L*. *mexicana species*	6 (8%)

Data expressed as n (%), unless otherwise specified.

N = Number of patients with available data for the respective category.

* Not further differentiated

Abbreviations: ACL, American Cutaneous Leishmaniasis; IIH, Charité Institute of International Health, Berlin, Germany; IQR, interquartile range.

Patients with complex lesions (32/75, 43%) had been exposed in Costa Rica (11/32, 34%), followed by Bolivia (6/32, 19%) and Peru (5/32, 16%). Complex lesions were most common among patients infected with *L*. *lainsoni* (3/3, 100%), followed by *L*. *braziliensis* (11/21, 52%), *L*. *panamensis* (8/16, 50%), and *L*. *guyanensis* (2/5, 40%), while patients with infections caused by *L*. *mexicana* (0/6) or *L*. *mexicana complex* (0/3) had simple lesions. Complex lesions were most common in patients traveling for work or education (13/22, 59%), followed by tourists (18/45, 40%) and immigrants or expats (1/7, 14%). The median duration of illness was the same (8 weeks) for patients with complex lesions or with simple lesions.

### Patient outcomes

After initial treatment, cure was the most common outcome (51/73, 70%), followed by treatment failure (10/73, 13%), relapse (7/73, 10%), and partial remission (5/73, 7%). Cure was more frequent in patients with simple lesions compared to patients with complex lesions (32/41, 78% vs. 19/32, 59%), in patients without lymphatic involvement compared to patients with lymphatic involvement (18/25, 72% vs. 11/20, 55%), and in patients without mucosal involvement compared to patients with mucosal involvement (48/69, 70% vs. 3/3, 50%). Adverse events after initial therapy were seen in 14/22 patients with this recorded outcome. Of these, serious adverse events occurred in 2 patients, one of whom developed reversible acute kidney injury after treatment with LAmB, the other patient developed a skin reaction with miltefosine. Three patients, two of which had recurrent disease, were lost to follow up after initiating treatment.

After secondary treatment, cure was the most common outcome as well (17/21, 80%), followed by relapse (2/21, 10%), and partial remission (1/21, 5%), or treatment failure (1/21, 5%). Among these patients, 6 experienced common adverse events, none of these serious.

### Species-specific therapy

Most patients received systemic therapy. The most commonly used drug was miltefosine (41/94, 45%), followed by LAmB (31/94, 33%), and systemic pentavalent antimony (s.p.A) (11/94, 12%). Cure rates according to pharmacotherapy used are shown in Table 2. In patients receiving systemic therapy, species-specific treatment recommendations of the European expert group “LeishMan” were followed during initial treatment in 30/45 (67%) patients and in 15/17 (88%) patients during a second therapeutic attempt. Cure was more frequently seen when recommendations of the “LeishMan” group were followed (33/45, 73%), compared to when systemic therapy did not follow their recommendations, (6/17, 35%; P = 0.008) (see Tables 3 and S1), with an unadjusted OR, 5.04 (95% CI; 1.58–17.64, model 1). The association persisted after adjustment for age, sex, previous therapy, complex lesions, and *Leishmania* species (adjusted OR, 5.06; 95% CI, 1.22–24.16, model 3), S2 Table.

**Table 2:** ACL cure rates according to the pharmacotherapeutic agent used of patients with imported ACL treated at the IIH in Berlin 2000–2023.

	Cure after initial therapy	Cure after secondary therapy	*Total cure*
**LAmB**	18/23 (79%)	6/8 (75%)	*24/31 (77%)*
**s.p.A.**	6/8 (75%)	3/3 (100%)	*9/11 (82%)*
**MLF**	21/33 (64%)	6/8 (75%)	*27/41 (66%)*
**Keto**	1/2 (50%)	-	*1/2 (50%)*
**Itra**	0/1 (0%)	-	*0/1 (0%)*
**p.l.A**	3/3 (100%)	2/2 (100%)	*5/5 (100%)*
**Paro**	1/2 (50%)	-	*1/2 (50%)*
**None**	1/1 (100%)	-	*1/1 (100%)*
*Total cure*	*51/73 (70%)*	*17/21 (81%)*	*68/94 (72%)*

Data as n/N (%)

Treatment outcomes were measured after three months. Cure was defined as re-epithelialization of ulcers or disappearance of papular-nodular lesions.

Abbreviations: ACL, American Cutaneous Leishmaniasis; IIH, Charité Institute of International Health, Berlin, Germany; s.p.A., systemic pentavalent antimony; MLF, miltefosine; LAmB, liposomal amphotericin B; Keto, Ketoconazole; Itra, itraconazole; p.l.A, perilesional pentavalent antimony; Paro; paromomycin

**Table 3:** ACL cure rates according to *Leishmania* species and systemic treatment in patients treated at IIH Berlin 2000–2023, N = 62. Species-specific treatments according to the “LeishMan” group recommendations are marked with an asterisk.

	Cure n/N (%)
***L*. *guyanensis species***	
MLF *	3/4 (75%)
LAmB	1/1 (100%)
s.p.A. *	1/1 (100%)
***L*. *panamensis species***	
MLF *	11/12 (91.7%)
LAmB	0/4 (0%)
s.p.A. *	1/2 (50%)
***L*. *braziliensis species***	
MLF (Bolivia or Brazil) §*	4/7 (57.1%)
MLF (not Bolivia or Brazil)	4/10 (40%)
LAmB *	5/8 (62.5%)
s.p.A. *	5/5 (100%)
***L*. *lainsoni species***	
MLF (Bolivia) §*	1/1 (100%)
LAmB *	2/2 (100%)
***L*. *mexicana species***	
MLF *	0/2 (0%)
Keto *	1/2 (50%)
Itraconazole	0/1 (0%)

Data expressed as n/N (%), n = number of complete remissions with the respective drug for the respective species, N = number of patients for whom the respective drug was used for the respective species.

Treatment outcomes were measured after three months. Cure was defined as re-epithelialization of ulcers or disappearance of papular-nodular lesions.

Cure after systemic therapy was seen in 33/45 (73.3%) patients receiving systemic therapy according to the “LeishMan” group recommendations, and in 6/17 (35.3%) of patients when these recommendations were not followed, P = 0.008.

* Species-specific treatment according to “LeishMan” group recommendations (1).

§ MLF therapy recommended only for patients exposed in Bolivia or Brazil

Abbreviations: ACL, American Cutaneous Leishmaniasis; IIH, Charité Institute of International Health, Berlin, Germany; s.p.A., systemic pentavalent antimony; MLF, miltefosine; LAmB, liposomal amphotericin B; Keto, Ketoconazole.

## Discussion

We conducted a retrospective analysis that included 75 patients with ACL presenting at our clinic in a period of over 20 years. The patients were mostly otherwise healthy young male adults, and the primary reason for travel was tourism. In this study, most of the infections were caused by *L*. *braziliensis*, the most prevalent species on the American continent, followed by *L*. *panamensis*. The most common travel destination in our cohort was Costa Rica, where *L*. *panamensis* is highly prevalent. Nearly half of our patients had complex lesions. Consequently, even though guidelines tend to favour localised therapy whenever possible to minimize toxicity risks and enhance patient acceptability [5,8,14,15], localised therapy was considered feasible in only a few cases.

Significant differences exist in the management approach in endemic compared to non-endemic countries, and current guidelines from PAHO/WHO are primarily tailored for endemic areas [7]. One difference is that in resource-limited settings, species identification is often challenging, although critical for targeted therapy and advocated by all guidelines [1,5,8]. It is known that the efficacy of drugs used to treat CL even varies for the same *Leishmania* species from different geographical regions [2]. Furthermore, systemic treatments in endemic areas may rely on more affordable, albeit more toxic, drugs. Systemic pentavalent antimonials remain the standard of care worldwide for CL in general and for ACL in particular, due to their availability and low cost, and despite frequent adverse events, drug resistance, and the necessity of parenteral administration [19,20]. Additionally, travellers from non-endemic regions might exhibit different immunological responses [2], a factor that may further lead to different responses to therapy. This underlines the need for treatment strategies that can cater to varying settings, species, severities and manifestations of ACL, including data on imported ACL [2].

In our cohort, cure was not achieved in approximately 30% of patients during a first therapeutic attempt, and in almost 20% of patients who underwent a second therapeutic attempt. A significant shift in our therapeutic approach was marked in 2014, aligning with the European expert group “LeishMan” recommendations for species-specific therapy [1]. This alignment allowed for a more nuanced treatment, improving patient outcomes. Our findings indicate that patients managed with species-specific therapy had roughly five times the odds of healing compared to patients who were treated not following these recommendations. Our findings provide additional evidence to a previous observation that found that in a cohort of patients with imported CL and muco-cutaneous leishmaniasis, species-specific therapy was associated with better outcomes [16].

In our cohort, the overall most efficacious systemic drug was IV pentavalent antimony. Pentavalent antimony was particularly efficacious in healing infections due to *L*. *braziliensis* that were acquired in Bolivia, Ecuador, Peru, Brazil, and Venezuela, respectively. LAmB was the second most efficacious drug and showed particular efficacy for healing infections caused by *L*. *lainsoni* acquired in Peru and Brazil, and it did not heal lesions caused by *L*. *panamensis* that were contracted in Costa Rica, confirming an already known lack of efficacy [1].

Treatment of CL with LAmB is mostly based on its efficacy in treating visceral leishmaniasis (VL). Consequently, the dosages are aligned to the VL regimen (total cumulative dose of 21mg/kg) [21], but the skin penetration of LAmB is not well studied and efficacy data for use of LAmB therapy for CL is primarily based on results from small, mostly observational studies that have shown various cure rates [22–26]. One recent retrospective cohort study in an *L*. *braziliensis* endemic area in Brazil compared the efficacy of LAmB with IV pentavalent antimony (meglumine antimoniate), and found that patients treated with meglumine antimoniate had a cure rate of 93.9% compared to a cure rate of 57.6% in the LAmB group [25]. In a recent systematic review of CL cases and case series primarily from South America that were treated with LAmB, an overall treatment efficacy of 82.3% was calculated [21].

Miltefosine was the most commonly used systemic drug in our cohort and the third most efficacious systemic drug for healing. It was particularly efficacious in the treatment of infections caused by *L*. *panamensis* acquired in Costa Rica, but not efficacious in the treatment of *L*. *mexicana* from Mexico. Miltefosine has previously shown good efficacy towards a wide range of *Leishmania* species causing ACL and Old World CL, and as an oral drug, it has the great advantage to be administered on an outpatient basis [27,28]. Data on the use of miltefosine for ACL is based mainly on studies in specific regions of and for species in Latin America, where efficacy has been variable, e.g., miltefosine for *L*. *braziliensis* from Guatemala had an efficacy of 48%, whereas in Brazil, the efficacy of miltefosine for *L*. *braziliensis* was 85% [28]. Although approved in Latin America in 2005, miltefosine is nowadays not commonly available in this and other leishmania-endemic areas [29].

Our results add to the existing literature on efficacy of treatment for different *Leishmania* species. Considering that ACL is a rare and non-notifiable imported disease in Germany, we have a limited number of cases for each species included. Therefore, the efficacy of the individual drugs used in our study should be interpreted with caution. To validate these findings conclusively, randomized controlled trials continue to be essential. Unfortunately, the rarity of such trials in the context of ACL, a neglected tropical disease, makes this a challenging prospect. Additionally, the difficulty of conducting controlled trials against different species further complicates the process.

Further limitations include the risk of information bias and residual confounding related to the retrospective nature. Additionally, due to missing data for this variable, we could not always accurately determine the proportion of patients who developed adverse effects to medication. Our single-site tertiary centre might also increase the risk of having a highly selected population (e.g., patients with more severe disease). Nevertheless, our study has several strengths, as all cases had a confirmed diagnosis of ACL with species identified in most, and all information regarding the therapeutic regimes was available. Furthermore, our results strengthen the evidence that species-specific systemic therapy is associated with an increased probability of healing, although not all CL species were represented in our sample.

## Conclusion

Our findings delineate ACL as an imported disease that is rarely seen in Germany. Almost half of our patients presented with complex lesions, challenging successful therapy. Our findings show that systemic therapy following the 2014 species-specific recommendations of the European expert group “LeishMan” was associated with better outcomes, independently of age, sex, previous therapy, complexity of the lesions, and species. This encouraging result highlights the importance of species identification to guide ACL management, and suggests that following a species-specific therapy approach may lead to better outcomes.

## Supporting information

S1 Fig Direct acyclic graphs (DAGs) that were used to obtain the set of variables to adjust for in the models. In S1A Fig, the DAG depicts the assumption that there is no direct effect of species on the outcome, i.e., cure, and therefore model 2 must not be adjusted for species. S1B Fig depicts a DAG that assumes a direct effect of species on the outcome and must therefore be adjusted for in model 3.
(TIF)

S1 Table Proportion of healing in patients with ACL after systemic therapy according to treatment that follows the “LeishMan” group recommendations. (DOCX)

S2 Table Odds ratios for cure after systemic species-specific therapy* among patients with imported ACL in patients treated at IIH Berlin 2000–2023. (DOCX)

S1 Data Raw data underlying this article. (XLSX)
