# Supplementary figures and images for: American Cutaneous Leishmaniasis: Imported cases in Berlin 2000–2023
Source: PLoS Negl Trop Dis. 2024 Jul 15;18(7):e0012323. doi: 10.1371/journal.pntd.0012323 (PMC11271916; doi:10.1371/journal.pntd.0012323)

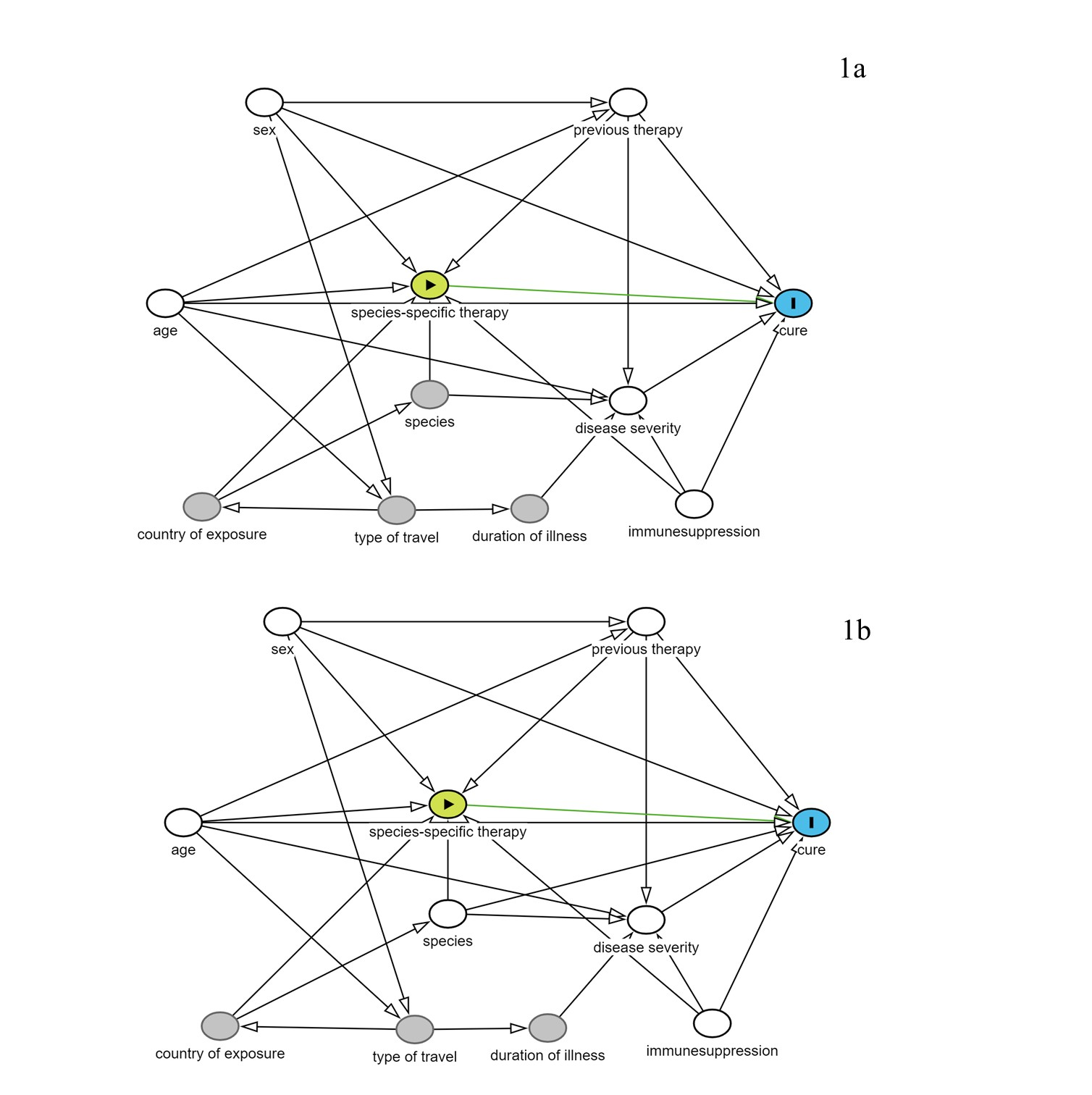

Supplement: S1 Fig — In S1A Fig, the DAG depicts the assumption that there is no direct effect of species on the outcome, i.e., cure, and therefore model 2 must not be adjusted for species. S1B Fig depicts a DAG that assumes a direct effect of species on the outcome and must therefore be adjusted for in model 3. (TIF) [file pntd.0012323.s001.tif]
