# Supplementary material for: American Cutaneous Leishmaniasis: Imported cases in Berlin 2000–2023
Source: PLoS Negl Trop Dis. 2024 Jul 15;18(7):e0012323. doi: 10.1371/journal.pntd.0012323 (PMC11271916; doi:10.1371/journal.pntd.0012323)
Supplement: S1 Table — (DOCX) [file pntd.0012323.s002.docx]

| **S1 Table. Proportion of healing in patients with ACL after systemic therapy according to treatment that follows the 2014 “LeishMan” group recommendations** | | | |
| --- | --- | --- | --- |
|  | Follows 2014 “LeishMan” recommendations for systemic therapy | |  |
|  | Yes | No | *P value* |
| Total, n/N (%) | 33/45 (73%) | 6/17 (35%) | *0.008* |
| - First attempt, n/N (%) | 20/30 (67%) | 6/15 (40%) | *0.1158* |
| - Second attempt, n/N (%) | 13/15 (87%) | 0/2 (0%) | *0.044* |
| Cure was more commonly seen in patients when the 2014 “LeishMan” group recommendations for systemic species-specific therapy were followed.  Prior to 2014, 19/29 (66%) systemic therapeutic attempts were aligned with what would later be recommended by the 2014 “LeishMan” group. During or after 2014, 26/33 (79%) systemic therapeutic attempts were in-line with the 2014 “LeishMan” group recommendations.  Abbreviations: ACL, American Cutaneous Leishmaniasis | | | |
