# Supplementary material for: American Cutaneous Leishmaniasis: Imported cases in Berlin 2000–2023
Source: PLoS Negl Trop Dis. 2024 Jul 15;18(7):e0012323. doi: 10.1371/journal.pntd.0012323 (PMC11271916; doi:10.1371/journal.pntd.0012323)
Supplement: S2 Table — (DOCX) [file pntd.0012323.s003.docx]

**S2 Table**

| **S2 Table. Odds ratios for cure after systemic species-specific therapy* among patients with imported ACL in patients treated at IIH Berlin 2000-2023** | | |
| --- | --- | --- |
|  | Unadjusted OR for cure (95% CI) | Adjusted ORs for cure (95% CI) |
| Model 1 | 5.04 (1.58- 17.64) | - |
| Model 2 | - | 4.93 (1.40-19.41) |
| Model 3 | - | 5.06 (1.22-24.17) |
| Model 1: unadjusted association with species-specific therapy as independent variable and cure as dependent variable. Model 2 included covariates of age, gender, previous therapy, and “complex lesions”§ as a measure of disease severity, which was the minimally sufficient confounder adjustment set according to our causal DAG, assuming no direct effect of *Leishmania* species on the outcome (S1 Fig, 1a). Model 3 included covariates of age, gender, previous therapy, Leishmania species, and “complex lesions”, which was the minimally sufficient adjustment set for d-separation between primary exposure and outcome according to our causal DAG, assuming that *Leishmania* species has a direct effect on the outcome (S1 Fig, 1b).  * As defined by the 2014 “LeishMan” group recommendations for species-specific therapy  § Complex lesions were defined as either ≥ 4 skin lesions, at least one skin lesion > 4 cm, or presence of mucosal involvement or lymphatic spread  Abbreviations: ACL, American Cutaneous Leishmaniasis; IIH, Charité Institute of International Health, Berlin, Germany; OR, odds ratio; CI, confidence interval; DAG, direct acyclic graph | | |
